# Development of prognostic signature based on immune-related genes in muscle-invasive bladder cancer: bioinformatics analysis of TCGA database

**DOI:** 10.18632/aging.103787

**Published:** 2021-01-19

**Authors:** Kun Jin, Shi Qiu, Di Jin, Xianghong Zhou, Xiaonan Zheng, Jiakun Li, Xinyang Liao, Lu Yang, Qiang Wei

**Affiliations:** 1 Department of Urology, Institute of Urology and National Clinical Research Center for Geriatrics, West China Hospital, Sichuan University, Chengdu, Sichuan, China; 2 Center of Biomedical Big Data, West China Hospital, Sichuan University, Chengdu, Sichuan, China

**Keywords:** muscle-invasive bladder cancer, The Cancer Genome Atlas, immune-related genes, prognostic signature, bioinformatics analysis

## Abstract

Background: Muscle-invasive bladder cancer (MIBC) with high tumor stages accounts for most bladder cancer patient mortality. Platinum-based chemotherapy provides insufficient survival benefits; however, immunotherapy is a promising option for MIBC.

Results: There were 31 differentially expressed IRGs that significantly correlated with the clinical outcomes of MIBC patients. A prognostic signature based on 12 IRGs (MMP9, RBP7, ADIPOQ, AHNAK, OAS1, RAC3, SLIT2, EDNRA, IL34, PDGFD, PPY, IL17RD) performed moderately in prognostic predictions with area under the curve (AUC) equal to 0.76. The high-risk patient group presented worse survival outcomes (hazard ratio 1.197, 95% confidence interval 1.103–1.299, *p* < 0.001). Furthermore, immune cell infiltration analysis showed increased tumor infiltration of macrophages in the high-risk group.

Conclusion: This novel prognostic signature can effectively divide MIBC patients into different risk groups, allowing for intensive treatment of high-risk individuals who have worse predicted survival outcomes.

Methods: Bioinformatics analyses were conducted using the Cancer Genome Atlas (TCGA) database. Differentially expressed genes and survival-associated immune-related genes (IRGs) were analyzed through a computational algorithm and Cox regression. The potential mechanisms of IRG expression were explored with transcription factors, and a prognosis classification based on IRG expression was developed to stratify patients into distinct risk groups.

## INTRODUCTION

Bladder cancer is the fifth most common cancer worldwide. There are approximately 76,960 new cases and 16,390 deaths per year in the United States [1]. Muscle-invasive bladder cancer (MIBC) with tumor stages T2 to T4 accounts for most patient mortality [2]. According to the European Association of Urology guidelines, radical cystectomy remains the standard primary treatment for MIBC [3]. Even though perioperative platinum-based chemotherapy improves overall survival compared with surgery alone [4], existing treatments for MIBC are insufficient because tumor recurrence and metastasis impede clinical management and decrease the survival of many patients. Therefore, careful monitoring of bladder cancer progression using specific and sensitive biomarkers could reduce the likelihood of advanced disease.

Immunotherapy has been a major driver of personalized medicine and has demonstrated aggressive anti-tumor effects by stimulating the immune system [5, 6]. Since immune components in the tumor microenvironment play an important role in tumor cell gene expression and clinical outcomes [7–9], measurement of these immune components provides a method of predicting the long-term prognosis of cancer patients. The differential expression of immune-related genes (IRGs) is considered an effective biomarker in many types of cancer.

This study explores IRG expression’s potential clinical utility for prognostic stratification and targeted MIBC immunotherapy. We analyzed IRG expression with corresponding clinical information to develop an individualized prognostic model for MIBC patients. Bioinformatics analyses were conducted to explore the underlying regulatory mechanisms of IRGs. Our results provide a foundation for developing personalized treatment for MIBC patients.

## RESULTS

### Identification of differentially expressed IRGs

**Figure 1:**
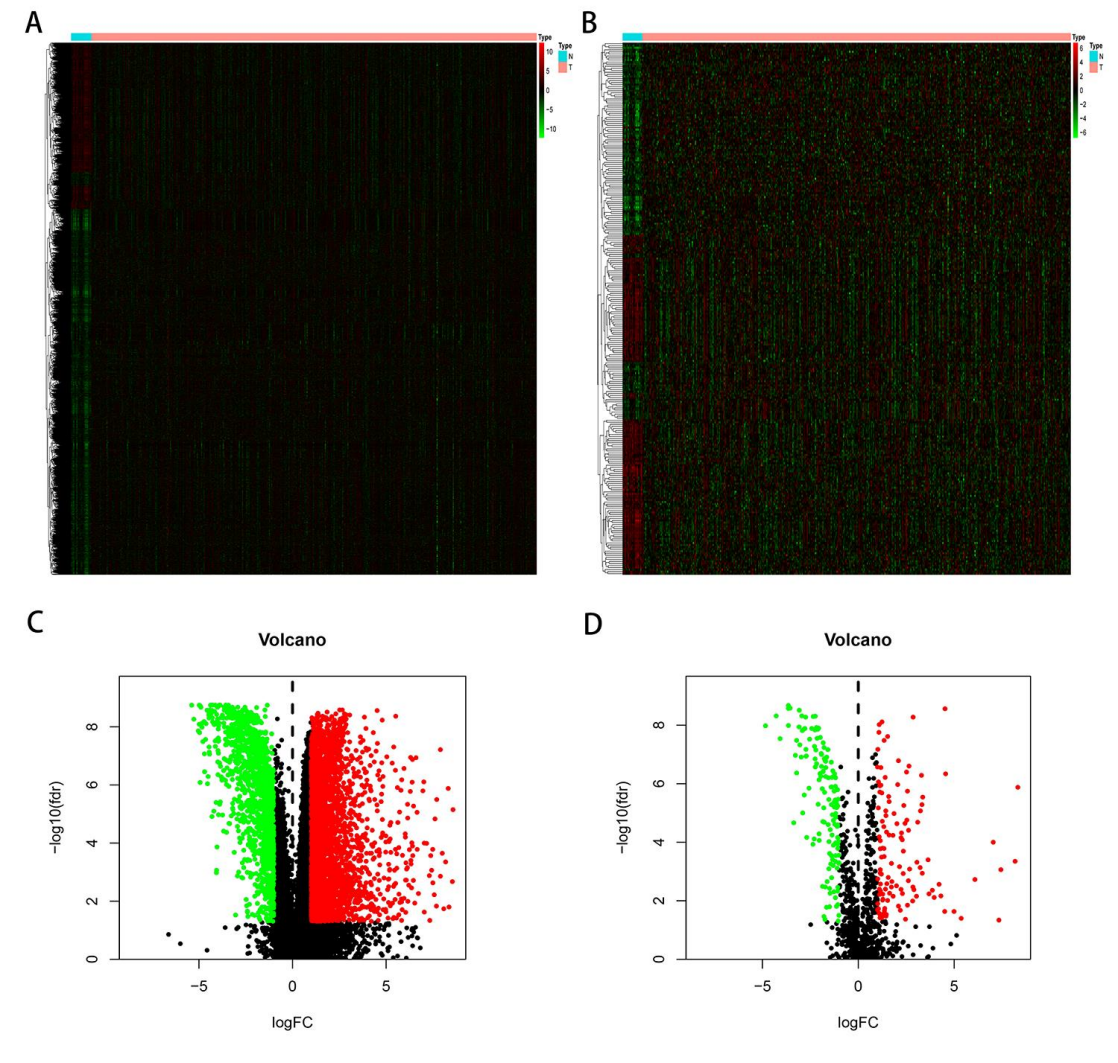
**Differentially expressed immune-related genes of bladder cancer from the TCGA database (TCGA-BLCA).** Heatmap (**A**) and volcano plot (**C**) demonstrating differentially expressed genes between bladder tumor and non-tumor tissues, green dots represent down-regulated expressed genes and red dots represent up-regulated expressed genes. Differentially expressed immune-related genes (IRGs) are shown in heatmap (**B**) and volcano plot (**D**), green dots represent down-regulated expressed genes and red dots represent up-regulated expressed genes (FDRfilter = 0.05, LogFCfilter = 1).

The edgeR algorithm identified 4,876 differentially expressed genes, of which 3,453 were upregulated and 1,423 downregulated (Figure 1A, 1C). From this set of genes, we extracted 260 differentially expressed IRGs, including 120 upregulated and 140 downregulated (Figure 1B, 1D). The list of differentially expressed genes and IRGs is presented in Supplementary Tables 1, 2, respectively.

**Figure 2:**
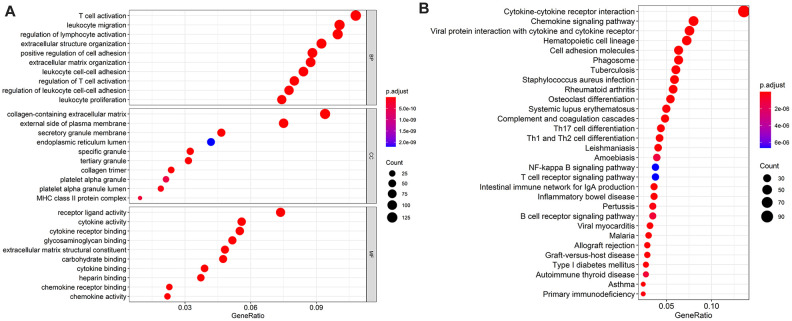
The top most 10 enriched Gene Ontology (GO) enrichment pathways in biological processes, cellular components, and molecular functions, respectively (**A**) and the top most 30 enriched Kyoto Encyclopedia of Genes and Genomes (KEGG) pathways (**B**). Notes: BP = biological processes; CC = cellular components; MF = molecular functions.

According to the GO enrichment analysis, inflammatory pathways were the most frequently enriched. “T cell activation,” “collagen-containing extracellular matrix,” and “receptor-ligand activity” were the most common terms among biological processes, cellular components, and molecular functions, respectively (Figure 2A). As for KEGG pathways, cytokine-cytokine receptor interactions were most often enriched by differentially expressed IRGs (Figure 2B).

### Identification of survival-associated IRGs

MIBC-specific IRGs related to survival outcomes (p < 0.01) were selected for the survival analysis to make the prognosis index more precise (Table 1). There were 31 IRGs that met this criterion, four of which were risk factors and the others protective factors.

**Table 1:** Characteristics of MIBC specific immune-related genes (univariate cox analysis, with p < 0.01).

**id**	**HR**	**HR.95L**	**HR.95H**	**pvalue**
THBS1	1.004	1.001	1.007	0.008
PI3	1.000	1.000	1.000	0.002
CXCL12	1.013	1.005	1.021	0.002
ZC3HAV1L	1.141	1.063	1.223	<0.001
MMP9	1.000	1.000	1.000	0.005
RBP7	1.014	1.007	1.020	<0.001
ADIPOQ	1.100	1.052	1.150	<0.001
ELN	1.016	1.004	1.028	0.009
PDGFRA	1.044	1.016	1.073	0.002
AHNAK	1.014	1.009	1.019	<0.001
PTX3	1.012	1.006	1.018	<0.001
IRF9	0.804	0.704	0.919	0.001
OAS1	0.978	0.967	0.988	<0.001
RAC3	1.026	1.014	1.037	<0.001
NFATC1	1.120	1.034	1.212	0.005
SLIT2	1.205	1.069	1.358	0.002
EDNRA	1.089	1.038	1.142	0.001
IGF1	1.376	1.212	1.563	<0.001
IL34	1.041	1.014	1.068	0.002
PDGFD	1.090	1.046	1.136	<0.001
PGF	1.034	1.016	1.052	<0.001
PPY	1.020	1.010	1.030	<0.001
ANGPTL1	1.026	1.006	1.045	0.009
GHR	1.232	1.052	1.443	0.010
IL17RD	1.068	1.019	1.120	0.006
NPR1	1.146	1.066	1.231	<0.001
NRP2	1.044	1.011	1.078	0.009
OXTR	1.034	1.008	1.060	0.010
PTGER3	1.284	1.078	1.530	0.005
TACR1	1.392	1.088	1.780	0.009
TGFBR2	1.015	1.004	1.026	0.006

### TF regulatory network

**Figure 3:**
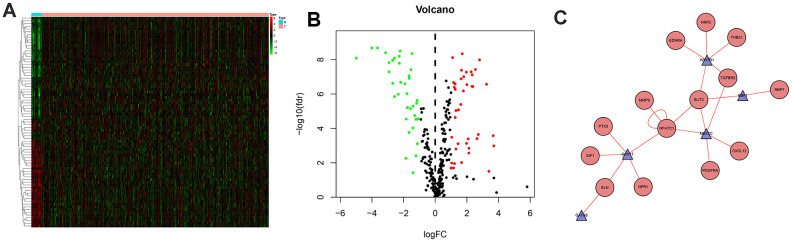
**Differentially expressed transcription factors and regulation network.** Heatmap (**A**) and volcano plot (**B**) showing different expressed transcription factors, green dots represent down-regulated and red dots represent up-regulated. And regulation network (**C**) presenting association of TFs and IRGs, red circles represent high risk genes and blue triangle represent TFs, while the red line represent up-regulated effects.

The regulatory relationships of the survival-associated IRGs and TFs were analyzed to explore the potential mechanisms corresponding to clinical significance (Figure 3). We examined the expression profiles of 318 TFs and found 77 that were expressed differently in MIBC than in normal tissues. Setting the correlation score cutoff value to > 0.5 resulted in 5 TFs that were screened to form the regulatory network in association with the IRGs.

### Evaluation of clinical outcomes

**Figure 4:**
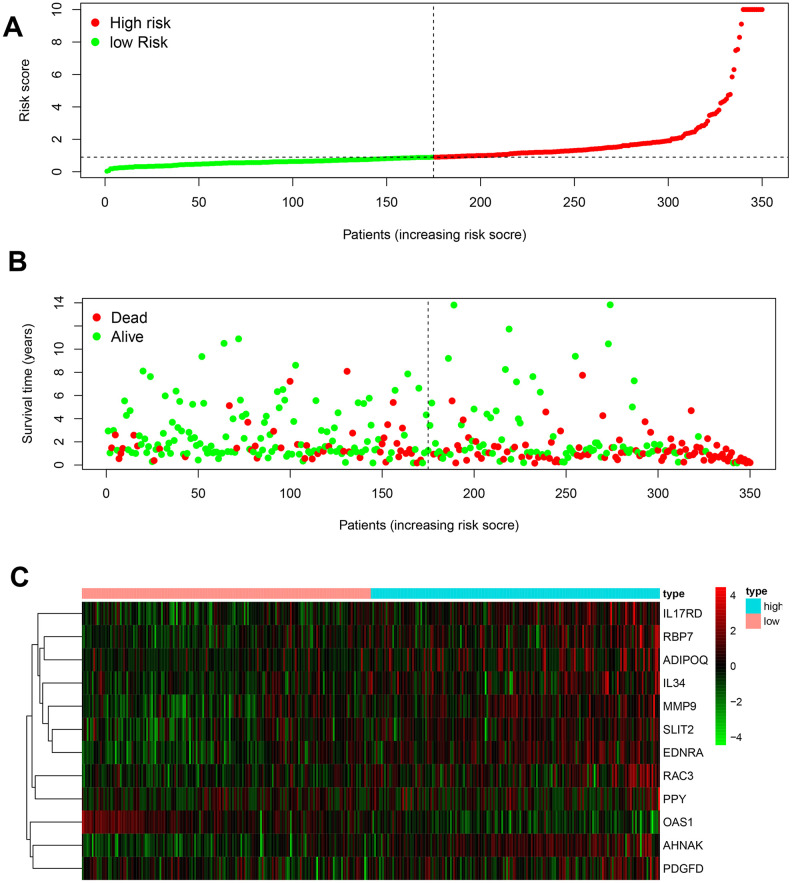
**Development of the prognostic index based on immune-related genes.** (**A**) Rank of prognostic index and distribution of groups. (**B**) Survival status of patients in different groups. (**C**) Heatmap of expression profiles of included genes.

Based on the LASSO regression analysis of the expression of IRGs, the patients were divided into two risk groups (Figure 4). The formula for the prognostic index was as follows:

(Expression level of MMP9 × 0.0004) + (Expression level of RBP7 × 0.0094) + (Expression level of ADIPOQ × 0.0800) + (Expression level of AHNAK × 0.0129) + (Expression level of OAS1 × −0.0163) + (Expression level of RAC3 × 0.0266) + (Expression level of SLIT2 × −0.1960) + (Expression level of EDNRA × 0.0829) + (Expression level of IL34 × 0.0290) + (Expression level of PDGFD × 0.0469) + (Expression level of PPY × 0.0210) + (Expression level of IL17RD × 0.0228).

**Figure 5:**
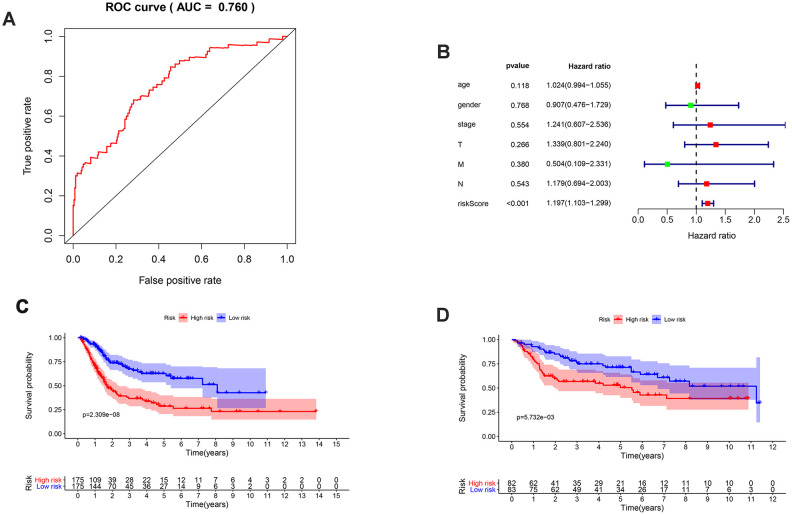
**Validation of the prognosis signature and survival outcomes of different risk groups.** (**A**) ROC curve of the prognosis signature with AUC equal to 0.760; (**B**) Multivariate Cox analysis of clinical parameters and risk score; (**C**) Kaplan-Meier survival curve of different risk groups of the TCGA database; (**D**) Kaplan-Meier survival curve of different risk groups of the GEO database in the form of cross validation.

With the AUC of the ROC curve equal to 0.760, the prognostic index showed moderate potential for prognosis prediction (Figure 5A). According to the results from the multivariate Cox regression, the prognostic risk score could act as an independent factor after adjusting for clinical parameters (hazard ratio 1.197, 95% confidence interval 1.103–1.299, *p* < 0.001] (Figure 5B). Overall survival of the low-risk group was significantly greater than the high-risk group (*p* < 0.001) based on the Kaplan-Meier survival curves (Figure 5C).

To further explore the impact of IRGs on clinical outcomes, we conducted multivariate regression of the IRGs to elucidate the relationships between IRG expression and the clinicopathological factors in MIBC (Table 2). Results showed patients in the high risk group presented higher T stage, M stage and clinical stage.

**Table 2:** Relationships between the expressions of the immune-related genes and the clinicopathological factors in MIBC.

**Genes**	**Age (>65/ ≤65)**		**Gender (Male/Female)**		**Clinical stage (Stage III-IV/Stage II)**		**T stage (T3-4/T2)**		**N stage (N1-3/N0)**		**M stage (M1/M0)**	
	**t**	**P**	**t**	**P**	**t**	**P**	**t**	**P**	**t**	**P**	**t**	**P**
MMP9	0.17	0.865	0.584	0.561	-1.533	0.128	-1.882	0.062	-0.959	0.371	-0.434	0.665
RBP7	-0.953	0.342	1.36	0.183	-2.313	0.022	-2.121	0.036	-1.636	0.153	-1.907	0.062
ADIPOQ	-1.022	0.308	1.047	0.302	-2.504	0.014	-2.498	0.014	-0.915	0.395	-1.469	0.148
AHNAK	-0.326	0.745	1.084	0.285	-3.822	<0.001	-4.049	<0.001	1.288	0.232	-2.263	0.027
OAS1	2.255	0.026	-2.568	0.012	2.339	0.022	2.349	0.021	3.367	0.009	2.103	0.038
RAC3	-0.43	0.668	1.2	0.237	-0.514	0.609	0.078	0.938	-1.619	0.156	-1.998	0.05
SLIT2	-0.917	0.361	1.076	0.288	-3.52	<0.001	-3.521	<0.001	-1.777	0.125	-1.933	0.057
EDNRA	-2.144	0.034	0.624	0.535	-4.102	<0.001	-4.271	<0.001	-1.825	0.114	-1.952	0.054
IL34	-0.124	0.901	1.506	0.14	-2.671	0.009	-2.722	0.008	-0.927	0.39	-1.99	0.052
PDGFD	1.259	0.212	0.519	0.607	0.184	0.855	0.198	0.843	-1.035	0.331	0.371	0.711
PPY	-1.208	0.229	-1.756	0.081	-1.704	0.091	-1.899	0.06	-0.784	0.46	1.234	0.219
IL17RD	-0.444	0.659	-1.231	0.221	-4.098	<0.001	-3.026	0.003	-0.738	0.485	-0.578	0.564
riskScore	-0.672	0.503	2.006	0.053	-3.252	0.001	-3.147	0.002	-1.297	0.242	-2.104	0.04

**Figure 6:**
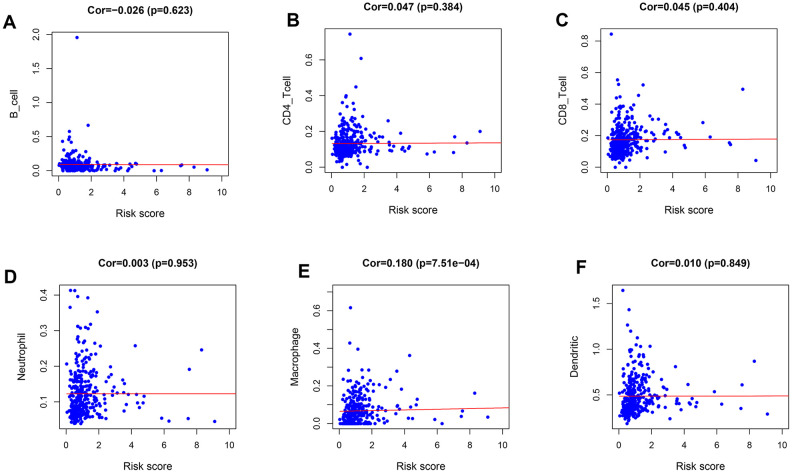
**Relationships between the immune-related prognostic index and infiltration abundances of six types of immune cells.** (**A**) B cells; (**B**) CD4 T cells; (**C**) CD8 T cells; (**D**) neutrophils; (**E**) macrophages; and (**F**) dendritic cells.

The immune cell infiltration analysis was performed to identify changes in the tumor microenvironment produced by the differential expression of IRGs (Figure 6). Among the six types of immune cells studied (B cells, CD4^+^ T cells, CD8^+^ T cells, macrophages, neutrophils, and dendritic cells), only macrophages showed a significant difference in infiltration abundance between the two risk groups (β = 0.18, *p* < 0.001).

## DISCUSSION

According to our bioinformatics analysis, 31 differentially expressed IRGs significantly correlated with the clinical outcome of MIBC patients. A prognostic signature based on 12 IRGs (MMP9, RBP7, ADIPOQ, AHNAK, OAS1, RAC3, SLIT2, EDNRA, IL34, PDGFD, PPY, IL17RD) performed moderately in prognostic predictions with AUC equal to 0.76. Patients in the high-risk group experienced worse survival outcomes compared with those in the low-risk group. Furthermore, immune cell infiltration analysis showed increased tumor infiltration of macrophages in the high-risk group, suggesting that an abnormal immunoreaction may have led to the disparate prognoses of this group.

Immune regulation plays an essential role in bladder cancer through intrinsic and extrinsic control of immune cell activation [10]. Tumor cells can exacerbate immunosuppressive pathways, fostering a tolerant microenvironment. The tumor microenvironment comprises many different immune cell subpopulations endowed with anti-tumor or pro-tumor activity [11]. Furthermore, different levels of tumor-infiltrating immune cells in the tumor microenvironment affect the prognosis of patients [12]. As for MIBC, molecular biomarkers and histopathology are predictive tools to assess possible clinical outcomes and guide further therapies [13, 14]. Based on these findings, IRG signatures could be used to identify potentially high-risk patients and thus allow for individualized treatment strategies.

Many of the 12 IRGs in our prognostic signature have previously been associated with cancer survival outcomes. Researchers have demonstrated that MMP9 is significantly associated with poor survival of cancer patients by way of promoting the invasiveness of cancer cells [15], and high expression of MMP9 has been observed in bladder cancer patients with poor prognosis and rapid tumor progression [16]. According to Elmasry et al., overexpression of RBP7 was an independent biomarker of poor cancer-specific survival in colon cancer [17], although no studies of RBP7 in MIBC have been reported. ADIPOQ is relevant in obesity and may potentially lead to metabolic syndrome [18], thus possibly worsening the prognosis of MIBC patients. As for AHNAK, two studies have conducted prognosis model analyses with this and other bladder cancer genes [19, 20], and both showed poor outcomes with high levels of AHNAK expression. One study has reported that the methylation status of SLIT2 is associated with low stage and histological grade for the initial diagnosis of non-MIBC [21]. Still, the gene expression level was unmentioned in the study. EDNRA overexpression was shown to be associated with metastasis and poor outcome in advanced bladder cancer patients [22]. Further research is needed to elucidate the impact of these IRGs on MIBC outcomes more fully.

Although several previous studies are focusing on distinct survival outcomes for differentially expressed genes [23–25], no genome-wide profiling studies of MIBC have been conducted. Until recently, the treatment of bladder cancer has made slow progress. Platinum-based chemotherapy is still widely used for metastatic bladder cancer without significant survival improvement [26]. However, in recent years, cancer immunotherapy has played an important role in clinical cancer management [27]. With the discovery of immune checkpoint inhibitors, MIBC patients may receive better prognoses. Programmed cell death 1 (PD-1, also known as PDCD1) on the surface of CD8^+^ T cells binds to programmed cell death ligand 1 (PD-L1, also known as CD274) produced by tumor tissue, leading to a limited host immune response. By increasing the level of infiltrated CD8^+^ T cells, PD-L1 inhibitors demonstrate an effective anti-tumor immune response [28]. T cells play an important role in the “immune surveillance” theories of cancer, with complex effects, including expression of polymorphic antigen receptors for specific antigen recognition, possession of effector functions, and development of memory characteristics [29, 30]. However, studies have only observed a significant increase in the level of CD4^+^ cells (specifically Th17 cells) in the blood of bladder cancer patients [31]; thus, the role of tumor-infiltrated CD4^+^ cells has not been elucidated. Our results showed no distinct expression differences of CD4^+^ cells in the two risk groups, indicating that the level of CD4^+^ cells probably does not independently reflect the risk classification for MIBC. Previous research demonstrated that macrophages are divided into different subtypes, with anti-tumor (M1) and pro-tumor activities (M2), but the holistic phenotype depended on the cytokine microenvironment in the tumor tissues [32]. For MIBC, high levels of tumor-infiltrated macrophages indicate a dismal prognosis and adjuvant chemotherapy resistance [33]. Our current analyses showed an upregulated level of macrophages in the high-risk group of MIBC patients. Moreover, Zhou et al. reported tumor-infiltrating neutrophils’ involvement in tumorigenesis and a negative correlation between neutrophils and CD8^+^ T cells [34], which indicates a state of immunosuppression in MIBC caused by a high level of tumor-infiltrating neutrophils.

Although a limitation of our study is that the effects of specific genes in tumor growth, invasion, and metastasis could not be evaluated, our analysis provided important information on MIBC survival outcomes.

## CONCLUSIONS

Our prognostic signature effectively divides MIBC patients into different risk groups; thus, individuals with worse predicted survival outcomes can be identified before the advancement of their disease and treated intensively with appropriate therapies. The differential gene expression identified in our study provides insight into the MIBC tumor microenvironment, contributing to the development of IRG-targeted therapies to improve the survival of MIBC patients.

## MATERIALS AND METHODS

### Clinical samples and data acquisition

We obtained transcriptome RNA-sequencing (RNA-seq) data of bladder cancer samples from the Cancer Genome Atlas (TCGA) database (https://portal.gdc.cancer.gov/projects/TCGA-BLCA), which included data from 414 primary cancers and 19 non-tumor tissues. Raw count data and relevant clinical information for the patients were also downloaded and extracted. A list of IRGs was obtained from the Immunology Database and Analysis Portal (ImmPort) database [35]. A list of 318 transcription factors (TFs) was downloaded from the Cistrome Cancer database [36] to explore the potential mechanisms of IRG expression. Patients with MIBC (tumor stage ≥T2) were identified with this clinical data and included in the study. Patients with a follow-up duration of less than 60 days were excluded. The analysis included 375 MIBC patients. To verify the robustness of the results, we used the Gene Expression Omnibus database to perform a cross-validation, among which 165 samples were selected. The flowchart outlining the sample selection is provided in Supplementary Figure 1.

### Differential gene analysis

We used the limma (linear models for microarray data) software package for the statistical R programming language to identify the differentially expressed genes between the tumor tissues and normal tissues [37], and the differential expressions were analyzed using the Wilcoxon test [38]. We set a false discovery rate <0.05 and a log2(fold change) >1 as the cutoff values. We then performed the Spearman correlation test to identify the association of IRGs and TFs, using a correlation filter of 0.5. Also, Gene Ontology (GO) enrichment was performed to investigate the functions of the gene products [39] and the Kyoto Encyclopedia of Genes and Genomes (KEGG) [40] pathway enrichment was conducted to explore the potential molecular mechanisms of the differentially expressed IRGs.

### Survival analysis and tumor-infiltrating immune cell analysis

Survival-associated IRGs for MIBC were identified using univariate and multivariate Cox regression analyses with a threshold value of *p* < 0.05. With the use of least absolute shrinkage and selection operator (LASSO) regression analysis, we selected specific IRGs. We calculated the individualized risk score with coefficients to construct a prognostic signature separating the high-risk and low-risk groups. Subsequently, Kaplan-Meier survival curves were generated based on the different risk groups to explore the survival differences. A receiver operating characteristic (ROC) analysis was performed to validate the predictive value of our model, and the area under the curve (AUC) was calculated. The Cox regression analyses were conducted for both clinical characteristics and risk scores to investigate whether risk score could independently predict survival prognosis. The tumor-infiltrating immune cell analysis (including B cells, CD4^+^ T cells, CD8^+^ T cells, macrophages, neutrophils, and dendritic cells) was performed using the TIMER database [41]. In the above analyses, *p* < 0.05 was considered statistically significant.

## Supplementary Material

Supplementary Figure 1

Supplementary Table 1

Supplementary Table 2
