# Supplementary figures and images for: Development of prognostic signature based on immune-related genes in muscle-invasive bladder cancer: bioinformatics analysis of TCGA database
Source: Aging (Albany NY). 2021 Jan 19;13(2):1859–71. doi: 10.18632/aging.103787 (PMC7880322; doi:10.18632/aging.103787)

## SUPPLEMENTARY FIGURE

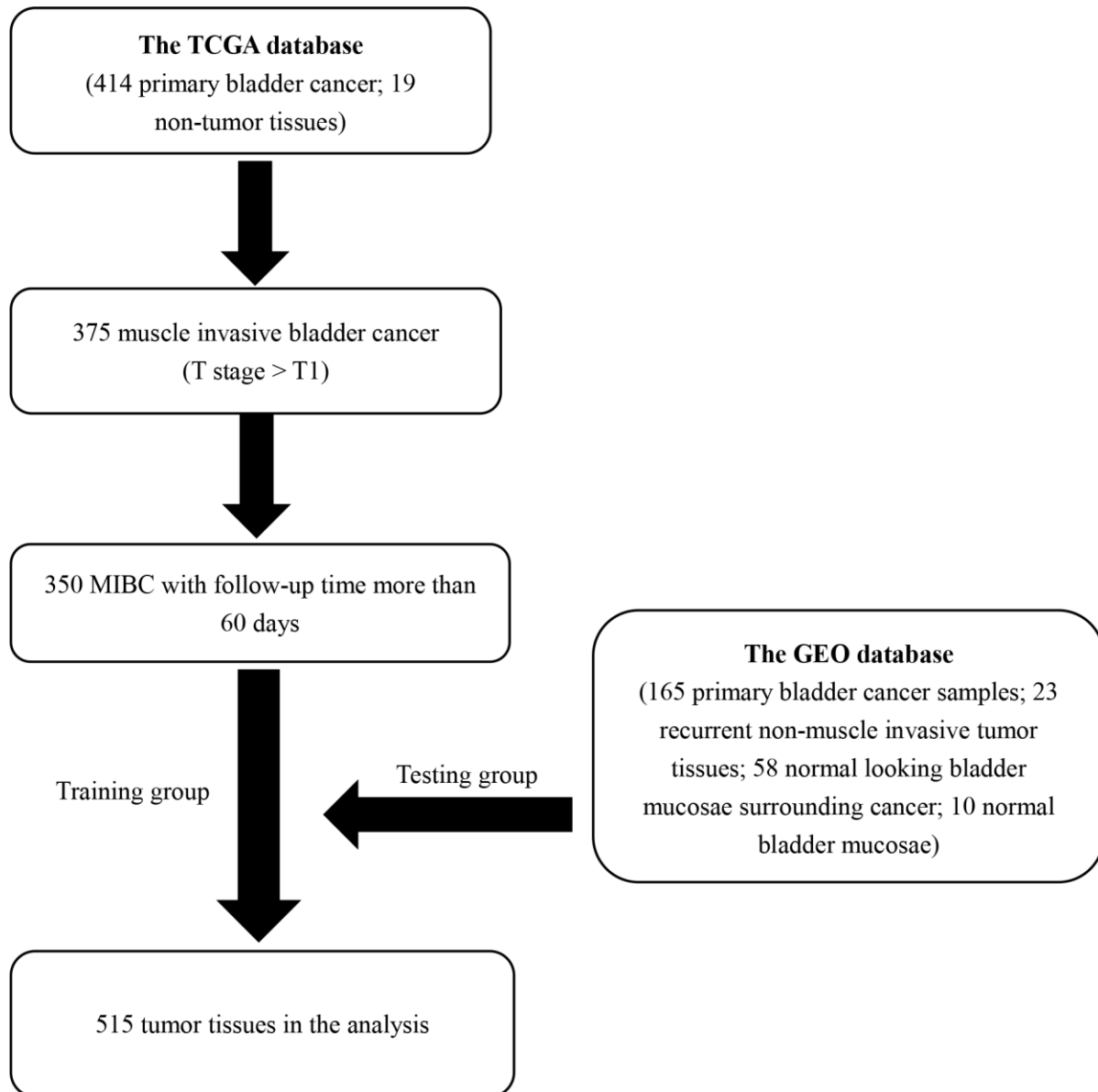

Supplementary Figure 1. Flowchart of sample selection.

Supplement: Supplementary Figure 1 [file aging-13-103787-s001.pdf]
